# Intention of healthcare providers to use video-communication in terminal care: a cross-sectional study

**DOI:** 10.1186/s12904-022-01100-5

**Published:** 2022-11-30

**Authors:** Richard M. H. Evering, Marloes G. Postel, Harmieke van Os-Medendorp, Marloes Bults, Marjolein E. M. den Ouden

**Affiliations:** 1 Research group Technology, Health & Care, Saxion, University of Applied Sciences, Enschede, Netherlands; 2 Research group Smart Health, Saxion, University of Applied Sciences, School of Health, Deventer/ Enschede, Netherlands; 3 Research group Care and Technology, Regional Community College of Twente, Hengelo, Netherlands

**Keywords:** Terminal care, Telemedicine, Videoconferencing, Intention

## Abstract

**Background:**

Interdisciplinary collaboration between healthcare providers with regard to consultation, transfer and advice in terminal care is both important and challenging. The use of video communication in terminal care is low while in first-line healthcare it has the potential to improve quality of care, as it allows healthcare providers to assess the clinical situation in real time and determine collectively what care is needed. The aim of the present study is to explore the intention to use video communication by healthcare providers in interprofessional terminal care and predictors herein.

**Methods:**

In this cross-sectional study, an online survey was used to explore the intention to use video communication. The survey was sent to first-line healthcare providers involved in terminal care (at home, in hospices and/ or nursing homes) and consisted of 39 questions regarding demographics, experience with video communication and constructs of intention to use (i.e. Outcome expectancy, Effort expectancy, Attitude, Social influence, Facilitating conditions, Anxiety, Self-efficacy and Personal innovativeness) based on the Unified Theory of Acceptance and Use of Technology and Diffusion of Innovation Theory. Descriptive statistics were used to analyze demographics and experiences with video communication. A multiple linear regression analysis was performed to give insight in the intention to use video communication and predictors herein.

**Results:**

90 respondents were included in the analysis.65 (72%) respondents had experience with video communication within their profession, although only 15 respondents (17%) used it in terminal care. In general, healthcare providers intended to use video communication in terminal care (*Mean (M)* = 3.6; Standard Deviation (*SD) =* .88). The regression model was significant (F = 9.809, *p*-value<.001) and explained 44% of the variance in intention to use video communication, with ‘Outcome expectancy’ (beta .420, *p* < .001) and ‘Social influence’ (beta .266, *p* = .004) as significant predictors.

**Conclusions:**

Healthcare providers have in general the intention to use video communication in interprofessional terminal care. However, their actual use in terminal care is low. ‘Outcome expectancy’ and ‘Social influence’ seem to be important predictors for intention to use video communication. This implicates the importance of informing healthcare providers, and their colleagues and significant others, about the usefulness and efficiency of video communication.

**Supplementary Information:**

The online version contains supplementary material available at 10.1186/s12904-022-01100-5.

## Background

Paliative care is provided to improve quality of life of patients who are diagnosed with an incurable and progressive illness, from which they will die within days up to several years. Palliative care includes disease and symptom oriented care, care in the dying phase, as well as bereavement and support of the family after death of the patient [1–3]. Terminal care is defined as the final phase of palliative care in which life expectancy is less than a few months and primarily focused on symptom relief [1, 4].

In the Netherlands, 72.7% of the patients who died at home received intensive care in the last 2 weeks of their life, i.e. additional care by general practitioners [5]. The general practitioner or specialist geriatric medicine, nurses and volunteers have a central role in this terminal care at home, hospices and nursing homes. Interprofessional collaboration for effective terminal care is both important and challenging [6–8]. Problems observed in terminal care are related to: 1) inaccurate adherence to disease-oriented management [9], 2) inadequate detection of symptoms and decisions within symptom management [10, 11], and 3) prescription of medication not in accordance with evidence-based practice [12–14]. These problems can lead to a restless dying process, with a major impact on patients and their loved ones [9, 12, 13, 15]. One of the causes of these problems is that involved healthcare providers are located at different locations, making it difficult to bring them together at the patients location to make joint decisions about symptom management [16]. It takes time to schedule and visit a patient, and sometimes an immediate decision or advice is desired.

Video communication has the potential to support collaboration between healthcare providers and patients in a simpler, faster and more efficient way [17]. Research on video communication among patients and healthcare providers in palliative care (including terminal care) shows promising results for a more timely detection of symptoms and symptom management by supporting remotely contact between healthcare providers and their patients [18–20]. The use of video communication among healthcare providers has increased during the Covid-19 pandemic, but still a substantial percentage of health care providers does not use it [21]. Even more, less is known about the specific use of video communication in terminal care [22]. More insight into reasons for use and non-use of video communication in terminal care can positively influence the implementation and thus improve the interprofessional communication in terminal care. Healthcare professionals prefer video communication over telephone contact for clinical assessments, as the quality is experienced as equivalent while video communication is more time-efficient [18, 20]. In addition, informal caregivers and patients are positive about the use of video communication, as it: 1) prevents distressing visits to the hospital, 2) makes it impossible to keep up a brave face, and 3) gives the patient a feeling of freedom to poor out one’s heart at a distance towards the healthcare provider [19]. Moreover, regular contact with the same group of healthcare providers improves the patients long-term engagement, improving access to healthcare professionals at home, enhanced feelings of security and safety, trustful relationships and fostered feelings of intimacy and relief [19, 21].

However, healthcare providers have mixed feelings about the use of video communication as difficulties are seen in the number of connection problems that cause interruptions in the conversation, and the inability in comforting the patient when talking about sensitive topics [19, 20]. Also other challenges are mentioned by healthcare providers for using video communication: 1) disrupted personal contact due to physical presence of other healthcare professionals in the same room, 2) hardware- and software breakdowns, 3) concerns about internet security and identity of the distant consultant, and 4) limited image quality for support of anamneses [19, 20]. The problems and challenges that healthcare providers face when applying video communication may decrease the intention to use video communication in terminal care. On the other hand, increased use of video communication may improve the collaboration between healthcare providers and a timely decision-making process with the patient [24, 25].

Implementation of video communication in terminal care may be accelerated if more is known about the relationship between intention to use video communication in terminal care and predictors herein. The Unified Theory of Acceptance and Use of Technology (UTAUT) is a widely recognized theoretical model describing predicting factors for intention to use of technology [26]. In addition, the Diffusion of Innovation theory (DOI) describes the role of personal innovativeness for the use of technology [27]. Based on the UTAUT and DOI, research has been conducted into the intention to use video communication in interprofessional terminal care and predictors herein. Hence, the research question of this study is: *“What is the intention of healthcare providers to use video communication in interprofessional terminal care and which predictors influence this intention?”*. It is expected that some of the healthcare providers do already use video communication within their work as healthcare provider (users) and some do not (non-users). A secondary research question is what the differences are between these two groups regarding intention to use and predictors herein.

## Methods

A cross-sectional study was performed among first-line healthcare providers working in The Netherlands for investigating the intention to use video communication in interprofessional terminal care and predictors herein, and what the differences are between ‘users’ and ‘non-users’.

### Procedures

An online survey (in Qualtrics) was set up and distributed via an anonymous link from October till December 2020. Participants were eligible if they worked in first-line healthcare (professionals and volunteers), i.e. care at home, hospices or nursing homes [28]. Participants were excluded from further analysis if they worked not directly as a healthcare provider. In the introductory text of the survey it was described that video communication can be used for contact between different healthcare providers located in different locations. Direct involvement of the patient in the conversation via video communication was not further described/required. The survey was distributed via professional organizations of general practitioners (THOON) and nurses (V&VN), general practitioners in the region Eemland, the association of palliative care (Stichting Leendert Vriel), WijkLink (learning platform in health and care) and network coordinators palliative care.

### Measurements and outcomes

The survey consisted of 39 questions divided into three parts: 1) demographic characteristics, 2) experience with video communication within the work as healthcare provider, 3) constructs about intention to use video communication within terminal care. The survey started with an information letter and consent form, followed by four questions about demographics. The second part started with the question: ‘*Do you have experience with video communication in your work as healthcare provider (yes/no)?*’. If so, follow-up questions were displayed regarding experience with different applications and functions. The second part ended with the question ‘*Do you use video communication also in terminal care (yes/no)?*’. The third part consisted of 31 items on constructs about intention to use video communication based on the Unified Theory of Acceptance and Use of Technology (UTAUT) and Diffusion of Innovation theory (DOI) [26, 27]. For a more detailed description of the constructs see table 1.

**Table 1:** Constructs ‘Intention to use’ and predictors hereof

Construct	Description	Number of items
Intention to use	The extent to which a person has the intention to use video communication in the upcoming 6 months.	3
Outcome expectancy	The extent to which a person finds the video communication useful and believes it will help them achieve their goals more efficiently.	3
Effort expectancy	The ease with which a person thinks they can apply video communication.	4
Attitude	A person’s affective response to the video communication	4
Social influence	The extent to which a person believes that colleagues and significant others encourage the use of video communication.	3
Facilitating conditions	The extent to which a person believes that the necessary knowledge and facilities for using video communication are in place.	4
Anxiety	The fear associated with the use of video communication.	3
Self-efficacy	Feelings of self-efficacy to deal with video communication.	3
Personal innovativeness	The willingness of persons to try out new ICT.	4

*The questions have been adapted from existing questionnaires* [29, 30]

For the correct completion of items in the third part, respondents were asked to consider their experience with video communication in the terminal care in the past 6 months, and if they had no experience with this, to fill out the items based on their own expectations about the use of it in the upcoming 6 months. The constructs included based on the UTAUT were: Intention to use (*n* = 3), Outcome expectancy (n = 3), Effort expectancy (*n* = 4), Attitude (n = 4), Social influence (n = 3), Facilitating conditions (n = 4), Anxiety (*n* = 3) and Self-efficacy (*n* = 3). Another validated construct about Personal innovativeness (4 items) based on DOI was added [27, 30, 31]. The wording of individual items were adapted to fit the research focus on video communication in terminal care. Items were scored on a standardized five point Likert scale from ‘1 = strongly disagree’ till ‘5 = strongly agree’. Scoring of three items were mirrored to improve reliability. In addition, the construct Anxiety had an inverse interpretation compared to the other constructs. Therefore, the mean construct score for Anxiety was recoded for unambiguous interpretation.

### Data processing and statistical analysis

The primary outcome measures were the construct scores for ‘intention to use’ and predictors hereof. The Cronbach’s alpha was calculated to check internal consistency of the constructs, which ranged from .446 to .919. One item in the construct Self-efficacy was taken out of the analysis as the Cronbach’s alpha increased from −.250 to .531. Because of that, 30 items about ‘Intention to use’ video communication and predictors herein were included in the analysis. Not all the constructs showed an high internal consistency. The constructs Facilitating conditions, Anxiety and Self-efficacy showed a low internal consistency (Cronbach’s alpha <.70). However, no other items were excluded as Cronbach’s alpha did not increase sufficiently. In addition, all constructs were kept in the analysis as these were based on validated questionnaires [29, 30].

Descriptive analysis of the demographics (age, gender, function, and healthcare institute) included calculation of frequencies, percentages, means (M) and standard deviation (SD) (Table 2). In visualizing the descriptive demographics of the sample, it was further subdivided into a group with experience in using video communication in their work as healthcare provider (users) and a group without this experience (non-users). Insight in the scores on the constructs about intention to use video communication and predictors hereof was provided per item by means of calculating the sum scores for each item.

**Table 2:** Characteristics of the respondents (*N* = 90)

	Total group (N = 90; 100%) n (%)
Mean age in years (SD)	45.8 (14.1)
Gender	
Female	78 (87%)
Male	12 (13%)
Profession	
Nurse	69 (77%)
Volunteer	9 (10%)
General practitioner	7 (8%)
Other^2^	5 (6%)
Healthcare institute^1^	
Home care	34 (38%)
Nursing home	30 (33%)
General practice	8 (9%)
Hospice – high care hospice	6 (7%)
Medical technical home care team	4 (4%)
Hospice – almost-home-home	2 (2%)
Hospital	1 (1%)
Other^3^	16 (18%)

^*1*^*Multiple answers per respondent were possible;* ^*2*^*nurse assistant (n = 3), occupational (n = 1) therapist, rehabilitation doctor (n = 1);* ^*3*^*e.g. rehabilitation center, small-scale housing, Extramural elderly care from a nursing home, care at home*

Mean construct scores of ‘Intention to use’ and predictors were calculated based on the individual item scores and ranged between 1 and 5. A higher score on the construct ‘Intention to use’ was indicative for an higher intention to use video communication, with a middle score of 3 indicative for a neutral/ inconclusive score. A higher score on a predictor was indicative of a more positive relationship with intention to use video communication, with also a middle score of 3 indicative for a neutral/ inconclusive score. The exemption was the predictor “anxiety”, for which a higher score was indicative for less anxiety, with a middle score of 3 indicative for a neutral score. Differences in mean construct scores between users and non-users were analyzed using an independent t-test (*p* < .05). If the Levene’s test for equality of variance was significant (p < .05) the Welch t-test was used. The Mann-Whitney U test was used instead of a t-test if the mean construct score was not normally distributed within each group.

Pearson correlation coefficients were used to analyze the strength of the bivariate correlation between ‘Intention to use’ and predictors (significance level *p* < .05). The correlation coefficients were interpreted as follows: <.30 as very low correlation; ≥.30 and < .50 as low correlation; ≥.50 and < .70 as moderate correlation; >.70 as high correlation.

A multiple linear regression analysis was performed to predict ‘Intention to use’ as the dependent variable (outcome), and the predictors as independent variables. A check on assumptions was performed for normal distribution of standardized residuals, and for linearity-, homoscedasticity- and multicollinearity of the regression model. All predictors were added (‘Enter’ method) because the model was theoretically substantiated. The ‘adjusted R^2^’ instead of ‘R^2^’ was used to determine the predictive value of the overall model in explaining the variance in ‘Intention to use’ as the number of respondents (*N* = 90) in the regression analysis was relatively low and the dependent variable ‘Intention to use’ was not normally distributed (*p* < .05 on the Shapiro-Wilk test of normality). The F-ratio was used for interpreting the significance (p < .05) of the adjusted R^2^. The Beta’s of the standardized coefficients were used for interpreting the contribution of each possible predictor in the model. Significance of each Beta was tested with a t-test (*p* < .10). The data were analyzed using SPSS, version 24.0 (IBM corp., Released 2016).

### Ethical considerations

This study was not subject to Medical Research Involving Human Subjects Act, as healthcare professionals were not asked to act or to change behaviors and the questions were not of a drastic nature [32–34].

## Results

**Fig. 1:**
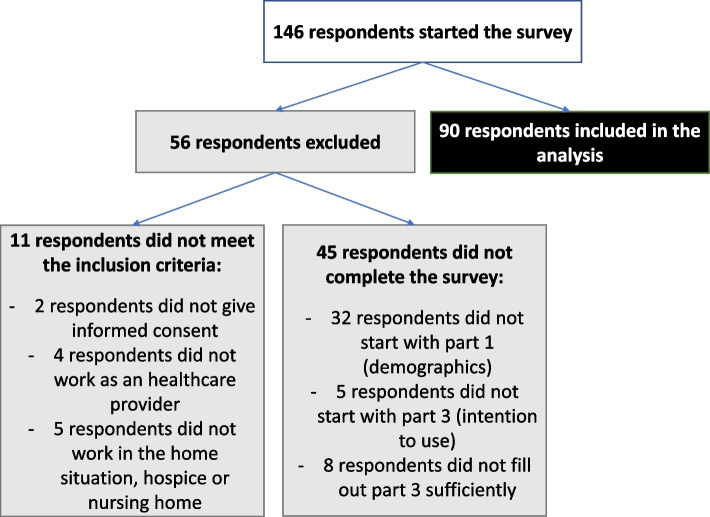
Flow diagram online survey

In total 146 respondents started with the survey, of which 90 respondents were included in the data-analysis (Fig. 1 flow diagram online survey).

### Demographic characteristics

The characteristics of the respondents are shown in table 2. Respondents were between 21 and 75 years. Most respondents had a background in nursing. Five respondents had another profession, i.e. nurse assistant (*n* = 3), occupational therapist (*n* = 1) and rehabilitation doctor (*n* = 1).

### Experience with use of video communication

65 (72%) respondents had experience with video communication in their work as healthcare provider (users) and 25 not (non-users). All volunteers were classified as non-users, as they indicated not to use video communication in their work as healthcare provider. The difference in mean (M) age between users of video communication (*M* = 42.7; *SD* = 12.2) and non-users (*M* = 54.0; *SD* = 15.7) was significant (*p* < .01). No other significant differences were found between users and non-users. 15 (17%) respondents had also experience with video communication in terminal care.

Among users (*n* = 65) the most frequent mentioned applications for video communication were Microsoft Teams (*n* = 46), Zoom (*n* = 33), WhatsApp (*n* = 26) and Skype (*n* = 11). Healthcare providers used video communication for group communication (*n* = 58), one-to-one communication (*n* = 54), video communication via smartphones (*n* = 32), chat function (*n* = 20), looking at a file together (*n* = 19) and saving and sharing files (*n* = 15).

### Intention to use video communication in interprofessional terminal care

Approximately 65% of the respondents ‘(strongly) agreed’ towards the items related to ‘Intention to use’ (intention to use: 61%; expectation to start: 63%, plan to use: 67%; see Additional file 1 appendix 1). This means that in general healthcare providers have the intention to use video communication in terminal care in the upcoming 6 months. However, about 14% of the respondents (strongly) disagreed towards items related to ‘Intention to use’ (intent: 14%; expect: 16%, plan to use: 12%; see Additional file 1 appendix 1).

The mean construct score (table 3) for ‘Intention to use’ was 3.6 (SD = 0.88), and this score was significant higher (*P* < .01) for users experienced with video communication in their work as healthcare provider (*M* = 3.8; *SD* = .78) vs. non-users (*M* = 2.9; *SD* = .84). The scores for predictors for intention to use indicated that most respondents (strongly) agreed with items related to ‘Effort expectancy’ (*M* = 3.7; *SD* = .76) and ‘Facilitating conditions’ (*M* = 3.6; SD = .54), and (strongly) disagreed with items related to ‘Anxiety’ (*M* = 3.7; *SD* = .60). The scores on items belonging to other predictors (‘Outcome expectancy’, ‘Attitude’, ‘Social influence’, ‘Self-efficacy’ and ‘Personal innovativeness’) were less conclusive with a substantial number of neutral scores at individual items and mean construct scores close to 3. The mean construct scores for ‘Social influence’, ‘Facilitating conditions’, ‘Anxiety’ and ‘Personal innovativeness’ were significantly higher for users experienced with video communication in their work as healthcare provider vs. non-users (*p*-values ranging from <.01 to <.05). Contrary, users scored significant lower for ‘Self-efficacy’ (*p* < .01).

**Table 3:** Mean (SD) construct scores for intention to use and possible predictors (*n* = 90)

Construct	Total group (n = 90) Mean (SD)	*Users (n = 65)* *Mean (SD)*	*Non-users (n = 25)* *Mean (SD)*	*p-values users* vs. *non users*
Intention to use^1^	3.6 (.88)	*3.8 (.78)*	*2.9 (.84)*	*<.001*
Outcome expectancy^1^	3.3 (.75)	*3.4 (.73)*	*3.1 (.79)*	*.088*
Effort expectancy^1^	3.7 (.76)	*3.8 (.73)*	*3.5 (.80)*	*.061*
Attitude^1^	3.3 (.71)	*3.4 (.72)*	*3.1 (.65)*	*.186*
Social influence^1^	3.1 (.65)	*3.2 (.65)*	*2.8 (.60)*	*.016*
Facilitating conditions^2^	3.6 (.54)	*3.7 (.52)*	*3.4 (.53)*	*.011*
Anxiety^1^	3.7 (.60)	*3.8 (.56)*	*3.4 (.60)*	*.002*
Self-efficacy^1^	3.1 (.82)	*2.9 (.85)*	*3.5 (.55)*	*.003*
Personal innovativeness^2^	3.3 (.75)	*3.5 (.76)*	*3.0 (.63)*	*.011*

^1^*Mann-Whitney U test and* ^2^*Independent t-test for testing differences between users* vs. *non-users*

### Correlation analysis and predictive model for intention to use

In table 4 the bivariate correlation coefficients (r) are shown for ‘Intention to use’ and potential predictors. ‘Intention to use’ had a moderate correlation with ‘Outcome expectancy’ (r = .608). All other potential predictors, except for ‘Self-efficacy’, had a low (≥.30 and < .50) or very low (<.30) correlation with ‘Intention to use’.

**Table 4:** Correlation coefficients and standardized coefficients of the independent variables (*n* = 90)

Independent variable	Pearson correlation coefficient r	*p*-values for r	Unstandardized coefficients B (SE)	Standardized coefficients Beta	*p*-values for Beta
Outcome expectancy	.608	<.001	.493 (.123)	.420^1^	<.001
Effort expectancy	.228	.015	.161 (.111)	.138	.153
Attitude	.487	<.001	.146 (.129)	.117	.260
Social influence	.450	<.001	.359 (.120)	.266^1^	<.004
Facilitating conditions	.369	<.001	.018 (.163)	.011	.910
Anxiety	.385	<.001	−.052 (.178)	−.035	.773
Self-efficacy	.010	.463	.102 (.101)	.094	.315
Personal innovativeness	.350	<.001	.125 (.110)	.106	.259

^1^*significant correlation with p < 0.01*

The adjusted R^2^ of the multiple linear regression analysis was .442 (F = 9.809, p < .001), meaning that all potential predictors together predicted 44% of the variation in the intention to use video communication. In table 4 the standardized coefficients (Beta’s) are shown for all potential predictors in the regression model. Only ‘Outcome expectancy’ and ‘Social influence’ significantly increased intention to use video communication in terminal care.

## Discussion

This study showed that the majority of healthcare providers have the intention to use video communication in the first-line terminal care, especially healthcare providers who have experience with video communication. ‘Outcome expectancy’ and ‘Social influence’ are significant predictors for intention to use video communication in terminal care. This means that the usefulness and efficiency of video communication in terminal care-, and the influence of colleagues and significant others-, are important predicting aspects for healthcare providers to actually use video communication in terminal care. These findings are in line with another study about the implementation of video communication in home-based palliative care [35]. Other studies found several barriers and factors diminishing the value proposition of video communication in palliative care [23, 35]. The reason for finding only predictors associated positively with the use of video communication in this study maybe due to the Covid-19 pandemic, as this study has been performed during the second wave of the Covid-19 pandemic in which the use of video communication has become a necessary good.

Most of the healthcare providers (72%) have already used video communication in their profession. However, the use of video communication in interprofessional terminal care is still relatively low (17%). This is especially true for volunteers, as none of them had already experience with video communication within their work as healthcare provider. However, even excluding volunteers, only 19% of healthcare professionals have experience with video communication in terminal care. This is striking as in other studies healthcare providers have indicated that they do see value in the use of video communication in terminal care as it is more time efficient, it can help to get a better assessment of a patient’s situation and it can improve interprofessional communication [24, 25, 36]. It is important to take into account that good quality of terminal care often benefits from the involvement of interprofessional collaboration between healthcare providers from different institutions [7, 8]. The use of video communication by different healthcare providers from different institutions may strengthen the interprofessional collaboration [37]. As a result of the Covid-19 pandemic the use of video communication in healthcare in general has increased substantially [22, 23, 38–41]. However, it is unclear whether the Covid-19 pandemic will lead to a sustainable embedding of video communication for interprofessional collaboration in terminal care.

Based on this study, in the implementation of video communication special attention should be paid towards current non-users of video communication. This sub group scored significant lower at intention to use video communication and at several predictors hereof (i.e. Social influence, Facilitating conditions, Anxiety, and Personal innovativeness). This is in accordance with another study in which healthcare providers experienced in the use of information and communication technology (like video communication) were more inclined to use it in diabetes management [42]. In this study non-users are also significantly elder, which is in line with the UTAUT model in which age and experience are moderators for the intention to use technology [26]. It is striking that current users of video communication scored lower on the construct ‘Self-efficacy’ than non-users. This may be explained by negative experiences that users have had with not being able to apply the technology themselves when using video communication [23, 35]. An additional difficulty in interprofessional terminal care is connecting healthcare providers from more than one organization. Safety regulations and the use of different applications often make it more difficult to have a video connection with someone from outside your own organization. An issue that current users may have encountered is network and bandwidth issues, especially when healthcare providers are at the patient’s home [43, 44]. Previous experiences with inappropriate use of video communication may also have damaged the self-confidence to use it properly [45].

Other important aspects in the implementation of video communication for interprofessional collaboration seem to be differences in profession and experience in working with technology. For instance, volunteers did not have any experience with video communication in their work as healthcare provider while most of the healthcare providers from other professions did so. Hence, in the implementation of video communication for interprofessional collaboration in terminal care it is important that factors related to the heterogeneity of healthcare providers are taken into account.

The strength of this study is the theoretical underpinning of the model used to predict intention to use video communication. 44% of the variation in the regression model for ‘Intention to use’ is predicted by the included factors. Maybe one or more variables are overlooked, like variables related to data security and ethical concerns as these aspects were not explicitly scored in this study but seen as part of the construct ‘Facilitating conditions’ [23]. Besides, the construct ‘Social influence’ did not include the opinion of patients and their families. Previous research showed that this had a major impact on health care providers’ judgments about the use of video communication in palliative care [35]. However, the focus of this study is primarily the use of video communication for interprofessional collaboration and therefore the opinion of patients and their families was not taken into account. A weakness of this study is the relative small group size, therefore the results should be interpreted carefully and additional subgroup analysis like in depth comparisons for respondents from different professions could not be performed. Another limitation is the distribution of the survey (online), and the recruitment of healthcare providers. Healthcare providers with a positive view regarding video communication or positive attitude toward use of technology could be overrepresented in the sample. Hence, this limits the external validity of the study.

## Conclusions

Healthcare providers have the intention to use video communication in terminal care, however actual use was low. In this study, outcome expectancy was an important predictor of intention to use video communication, which underlines the importance of informing healthcare providers about the usefulness and efficiency of video communication. Social influence was also a significant predictor, indicating that support by colleagues and significant others is important as well. Healthcare providers without experience with video communication within their work as healthcare provider had a significant lower intention to use video communication in terminal care. Therefore, it is recommended to pay special attention towards these inexperienced healthcare providers when implementing video communication for interprofessional collaboration in terminal care.

## Supplementary Information

**Additional file 1:** **Appendix 1.** Results part 3 of the survey

## Data Availability

The anonymous research data (including during data processing) is stored on secured server designed for research. The datasets generated and/or analysed during the current study are available in the DANS repository (10.17026/dans-xnv-ghwx). When making the data open access, it was decided to clean up the raw data by omitting answers to open questions, since in theory this information makes it possible to discover the identity of respondents. The data processing method is available upon request by contacting the first author (Richard Evering, r.m.h.evering@saxion.nl).

## Abbreviations

DOI: Diffusion of Innovation theory
UTAUT: Unified Theory of Acceptance and Use of Technology
