# Supplementary material for: Intention of healthcare providers to use video-communication in terminal care: a cross-sectional study
Source: BMC Palliat Care. 2022 Nov 30;21:213. doi: 10.1186/s12904-022-01100-5 (PMC9713136; doi:10.1186/s12904-022-01100-5)
Supplement: Supplementary file 1 — Additional file 1: Appendix 1. Results part 3 of the survey [file 12904_2022_1100_MOESM1_ESM.docx]

Appendix 1: Results part 3 of the survey

|  | **Total group**  **(N=90)**  **N (%)** | ***Users***  ***(n=65)***  ***N (%)*** | ***Non-users***  ***(n=25)***  ***N (%)*** |
| --- | --- | --- | --- |
| **Intention to use** |  |  |  |
| *“I intend to use online video communication in the upcoming 6 months.”* | | | |
| (Strongly) disagree (score 1-2) | 13 (14%) | *6 (9%)* | *7 (28%)* |
| Neutral (score 3) | 22 (24%) | *12 (18%)* | *10 (40%)* |
| (Strongly) agree (score 4-5) | 55 (61%) | *47 (72%)* | *8 (32%)* |
| *“I expect to start using online video communication in the upcoming 6 months.”* | | | |
| (Strongly) disagree (score 1-2) | 14 (16%) | *5 (8%)* | *9 (36%)* |
| Neutral (score 3) | 19 (21%) | *10 (15%)* | *9 (36%)* |
| (Strongly) agree (score 4-5) | 57 (63%) | *50 (77%)* | *7 (28%)* |
| *“I plan to use online video communication in the upcoming 6 months.”* | | | |
| (Strongly) disagree (score 1-2) | 11 (12%) | *5 (8%)* | *6 (24%)* |
| Neutral (score 3) | 19 (21%) | *9 (14%)* | *10 (40%)* |
| (Strongly) agree (score 4-5) | 60 (67%) | *51 (78%)* | *9 (36%)* |
| **Outcome expectancy** |  |  |  |
| *“I find online video communication useful in terminal care.”* | | | |
| (Strongly) disagree (score 1-2) | 12 (13%) | *9 (14%)* | *3 (12%)* |
| Neutral (score 3) | 30 (33%) | *19 (29%)* | *11 (44%)* |
| (Strongly) agree (score 4-5) | 48 (53%) | *37 (57%)* | *11 (44%)* |
| *“Using online video communication allows me to complete my tasks faster.”* | | | |
| (Strongly) disagree (score 1-2) | 15 (17%) | *10 (15%)* | *5 (20%)* |
| Neutral (score 3) | 33 (37%) | *22 (34%)* | *11 (44%)* |
| (Strongly) agree (score 4-5) | 42 (47%) | *33 (51%)* | *9 (36%)* |
| *“Using online video communication increases my productivity.”* | | | |
| (Strongly) disagree (score 1-2) | 20 (22%) | *11 (17%)* | *9 (36%)* |
| Neutral (score 3) | 40 (44%) | *31 (48%)* | *9 (36%)* |
| (Strongly) agree (score 4-5) | 30 (33%) | *23 (26%)* | *7 (28%)* |
| **Effort expectancy** |  |  |  |
| *“Handling online video communication is clear and understandable to me.”* | | | |
| (Strongly) disagree (score 1-2) | 6 (7%) | *3 (5%)* | *3 (12%)* |
| Neutral (score 3) | 14 (16%) | *8 (12%)* | *6 (24%)* |
| (Strongly) agree (score 4-5) | 70 (78%) | *54 (83%)* | *16 (64%)* |
| *“Becoming proficient in using online video communication is easy for me.”* | | | |
| (Strongly) disagree (score 1-2) | 10 (11%) | *7 (11%)* | *3 (12%)* |
| Neutral (score 3) | 11 (12%) | *8 (12%)* | *3 (12%)* |
| (Strongly) agree (score 4-5) | 69 (77%) | *50 (77%)* | *19 (76%)* |
| *“Online video communication is effortless for me to handle.”* | | | |
| (Strongly) disagree (score 1-2) | 16 (18%) | *11 (17%)* | *5 (20%)* |
| Neutral (score 3) | 21 (23%) | *12 (18%)* | *9 (36%)* |
| (Strongly) agree (score 4-5) | 53 (59%) | *42 (65%)* | *11 (44%)* |
| *“Learning to use online video communication is easy for me.”* | | | |
| (Strongly) disagree (score 1-2) | 12 (13%) | *6 (9%)* | *6 (24%)* |
| Neutral (score 3) | 13 (14%) | *7 (11%)* | *6 (24%)* |
| (Strongly) agree (score 4-5) | 65 (72%) | *52 (80%)* | *13 (52%)* |
| **Attitude** |  |  |  |
| *“Using online video communication is a bad idea.”* | | | |
| (Strongly) disagree (score 1-2) | 74 (82%) | *55 (85%)* | *19 (76%)* |
| Neutral (score 3) | 12 (13%) | *7 (11%)* | *5 (20%)* |
| (Strongly) agree (score 4-5) | 4 (4%) | *3 (5%)* | *1 (4%)* |
| *“Online video communication makes work more interesting.”* | | | |
| (Strongly) disagree (score 1-2) | 27 (30%) | *20 (31%)* | *7 (28%)* |
| Neutral (score 3) | 37 (41%) | *24 (37%)* | *13 (52%)* |
| (Strongly) agree (score 4-5) | 26 (29%) | *21 (32%)* | *5 (20%)* |
| *“Working with online video communication is fun.”* | | | |
| (Strongly) disagree (score 1-2) | 23 (26%) | *17 (26%)* | *6 (24%)* |
| Neutral (score 3) | 35 (39%) | *23 (35%)* | *12 (48%)* |
| (Strongly) agree (score 4-5) | 32 (36%) | *25 (38%)* | *7 (28%)* |
| *“I like working with online video communication.”* | | | |
| (Strongly) disagree (score 1-2) | 18 (20%) | *11 (17%)* | *7 (28%)* |
| Neutral (score 3) | 41 (46%) | *29 (45%)* | *12 (48%)* |
| (Strongly) agree (score 4-5) | 31 (34%) | *25 (38%)* | *6 (24%)* |
| **Social influence** |  |  |  |
| *“People who influence me think I should use online video communication.”* | | | |
| (Strongly) disagree (score 1-2) | 26 (29%) | *19 (29%)* | *7 (28%)* |
| Neutral (score 3) | 40 (44%) | *26 (40%)* | *14 (56%)* |
| (Strongly) agree (score 4-5) | 24 (27%) | *20 (31%)* | *4 (16%)* |
| *“People who are important to me think that I should use online video communication.”* | | | |
| (Strongly) disagree (score 1-2) | 27 (30%) | *18 (28%)* | *9 (36%)* |
| Neutral (score 3) | 44 (49%) | *32 (49%)* | *12 (48%)* |
| (Strongly) agree (score 4-5) | 19 (21%) | *15 (23%)* | *4 (16%)* |
| *“In general, the colleagues I work with support the use of online video communication.”* | | | |
| (Strongly) disagree (score 1-2) | 15 (17%) | *7 (11%)* | *8 (32%)* |
| Neutral (score 3) | 23 (26%) | *14 (22%)* | *9 (36%)* |
| (Strongly) agree (score 4-5) | 52 (58%) | *44 (68%)* | *8 (32%)* |
| **Facilitating conditions** |  |  |  |
| *“I have the necessary resources to make use of online video communication.”* | | | |
| (Strongly) disagree (score 1-2) | 8 (9%) | *4 (6%)* | *4 (16%)* |
| Neutral (score 3) | 14 (16%) | *11 (17%)* | *3 (12%)* |
| (Strongly) agree (score 4-5) | 68 (76%) | *50 (77%)* | *18 (72%)* |
| *“I have the necessary knowledge to use online video communication.”* | | | |
| (Strongly) disagree (score 1-2) | 8 (9%) | *4 (6%)* | *4 (16%)* |
| Neutral (score 3) | 13 (14%) | *7 (11%)* | *6 (24%)* |
| (Strongly) agree (score 4-5) | 69 (77%) | *54 (83%)* | *15 (60%)* |
| *“Online video communication does not fit into my work process.”* | | | |
| (Strongly) disagree (score 1-2) | 58 (64%) | *48 (74%)* | *10 (40%)* |
| Neutral (score 3) | 22 (24%) | *12 (18%)* | *10 (40%)* |
| (Strongly) agree (score 4-5) | 10 (11%) | *5 (8%)* | *5 (5%)* |
| *“I have a specific person (or service) available for help with problems/support with online video communication.”* | | | |
| (Strongly) disagree (score 1-2) | 27 30%) | *19 (29%)* | *8 (32%)* |
| Neutral (score 3) | 18 (20%) | *11 (17%)* | *7 (28%)* |
| (Strongly) agree (score 4-5) | 45 (50%) | *35 (54%)* | *10 (40%)* |
| **Anxiety** |  |  |  |
| “I am reluctant to use online video communication.” | | | |
| (Strongly) disagree (score 1-2) | 47 (52%) | *40 (62%)* | *7 (28%)* |
| Neutral (score 3) | 25 (28%) | *16 (25%)* | *9 (36%)* |
| (Strongly) agree (score 4-5) | 18 (20%) | *9 (14%)* | *9 (36%)* |
| *“I am afraid of making mistakes when using online video communication.”* | | | |
| (Strongly) disagree (score 1-2) | 71 (79%) | *53 (82%)* | *18 (72%)* |
| Neutral (score 3) | 11 (12%) | *8 (12%)* | *3 (12%)* |
| (Strongly) agree (score 4-5) | 8 (9%) | *4 (6%)* | *4 (16%)* |
| *“Online video communication feels somewhat intimidating to me.”* | | | |
| (Strongly) disagree (score 1-2) | 73 (81%) | *57 (88%)* | *16 (64%)* |
| Neutral (score 3) | 11 (12%) | *6 (9%)* | *5 (20%)* |
| (Strongly) agree (score 4-5) | 6 (7%) | *2 (3%)* | *4 (16%)* |
| **Self-efficacy** |  |  |  |
| *“I can work with online video communication, provided I can call on someone for help when I get stuck.”* | | | |
| (Strongly) disagree (score 1-2) | 37 (41%) | *32 (49%)* | *5 (20%)* |
| Neutral (score 3) | 20 (22%) | *14 (22%)* | *6 (24%)* |
| (Strongly) agree (score 4-5) | 33 (37%) | *19 (29%)* | *14 (56%)* |
| *“I can work with online video communication, provided I have enough time to complete my work.”* | | | |
| (Strongly) disagree (score 1-2) | 22 (24%) | *20 (31%)* | *2 (8%)* |
| Neutral (score 3) | 21 (23%) | *15 (23%)* | *6 (24%)* |
| (Strongly) agree (score 4-5) | 47 (52%) | *30 (46%)* | *17 (68%)* |
| **Personal innovativeness** |  |  |  |
| *“When I hear about a new technology, I want to try it out.”* | | | |
| (Strongly) disagree (score 1-2) | 12 (13%) | *6 (9%)* | *6 (24%)* |
| Neutral (score 3) | 31 (34%) | *24 (37%)* | *7 (28%)* |
| (Strongly) agree (score 4-5) | 47 (52%) | *35 (54%* | *12 (48%)* |
| *“In general, I am reluctant to try new technologies.”* | | | |
| (Strongly) disagree (score 1-2) | 56 (62%) | *46 (71%)* | *10 (40%)* |
| Neutral (score 3) | 20 (22%) | *13 (20%)* | *7 (28%)* |
| (Strongly) agree (score 4-5) | 14 (16%) | *6 (9%)* | *8 (32%)* |
| *“Compared to the people around me, I'm usually one of the first to try out new technologies.”* | | | |
| (Strongly) disagree (score 1-2) | 38 (42%) | *25 (38%)* | *13 (52%)* |
| Neutral (score 3) | 27 (30%) | *20 (31%)* | *7 (28%)* |
| (Strongly) agree (score 4-5) | 25 (28%) | *20 (31%)* | *5 (20%)* |
| *“I like to try out new technology.”* | | | |
| (Strongly) disagree (score 1-2) | 17 (19%) | *9 (14%)* | *8 (32%)* |
| Neutral (score 3) | 24 (27%) | *16 (25%)* | *8 (32%)* |
| (Strongly) agree (score 4-5) | 49 (54%) | *40 (62%)* | *9 (36%)* |
